# Genotyping by Amplicon Sequencing (GBAS) With Newly Developed SSR and EPIC Markers Reveals Structure in Populations of the Green Toad (*Bufotes viridis*) Across Rural and Urban Environments

**DOI:** 10.1002/ece3.71652

**Published:** 2025-07-08

**Authors:** Vincent Kendlbacher, Yoko Philipina Krenn, Thapasya Vijayan, Christina Rupprecht, Magdalena Spießberger, Manuel Curto, Lukas Landler, Harald Meimberg

**Affiliations:** ^1^ Department of Ecosystem Management, Climate and Biodiversity, Institute of Integrative Nature Conservation Research BOKU University Vienna Austria; ^2^ Department of Ecosystem Management, Climate and Biodiversity, Institute of Zoology BOKU University Vienna Austria; ^3^ CIBIO—Research Center in Biodiversity and Genetic Resources Vairão Portugal; ^4^ BIOPOLIS Program in Genomics, Biodiversity and Land Planning CIBIO Vairão Portugal

**Keywords:** amphibian, conservation genetics, genetic markers, population genetics, urban environment

## Abstract

Microsatellites (SSRs) are reliable markers for population genetic analyses but often suffer from null alleles caused by mutations in primer binding sites. Exon‐primed intron‐crossing (EPIC) markers address these limitations and serve as a complementary tool. In this study, we developed SSR and EPIC markers for the green toad ( *Bufotes viridis* ). We demonstrated their effectiveness using genotyping by high‐throughput amplicon sequencing (GBAS) across urban and rural populations. Marker development followed an established in‐house protocol. We then tested their functionality in singleplex‐PCRs before employing them in multiplex reactions and preparing them for sequencing. We analyzed the resulting data using several variability measures as well as individual‐based clustering methods. From the initial set of 48 markers of each type, 35 SSR and 46 EPIC markers consistently amplified across samples both in singleplex and multiplex assays. Data analysis did not corroborate the expectation of a continuous reduction of diversity in urban populations compared to rural ones. Clustering methods using EPIC markers revealed ecologically coherent results, showing weaker genetic structure in rural environments, whereas the SSRs' signal reflected drift‐induced patterns. Our findings suggest that the green toad exhibits a degree of resilience to urban environments. Furthermore, EPIC markers not only complement SSRs by having fewer null alleles but also provide greater robustness to random drift events. We recommend their combined use, especially in fragmented environments prone to genetic drift, such as urban areas.

## Graphical abstract

We developed and tested SSR and EPIC markers for the green toad ( *Bufotes viridis* ), demonstrating their effectiveness using genotyping by high‐throughput amplicon sequencing (GBAS) across urban and rural populations. EPIC markers exhibited fewer null alleles and provided more ecologically coherent clustering results, while SSRs reflected drift‐induced patterns. Our findings indicate that *B. viridis* may retain genetic diversity in urban environments and highlight the value of combining EPIC and SSR markers, particularly in fragmented habitats prone to genetic drift.

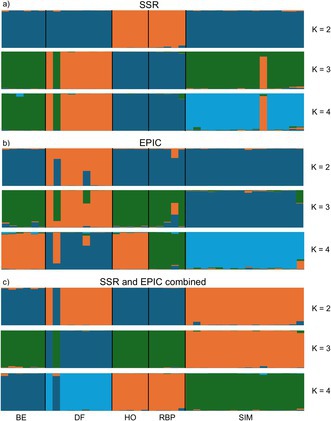

## 1 Introduction

Microsatellites or simple sequence repeats (SSRs) are repetitive segments of typical one to six nucleotides. The number of repetitions is highly variable and can reach several dozen (Selkoe and Toonen 2006). Replication slippage, the mechanism generating variation in the repeat number, is responsible for the comparably high mutation rate (Ellegren 2004; Li et al. 2002). SSRs have proven to be particularly reliable markers for population genetic analyses (Guichoux et al. 2011; Selkoe and Toonen 2006; Tautz and Schlötterer 1994). They are ubiquitous throughout the eukaryotic genome, follow Mendelian co‐dominant inheritance, and, owing to their increased mutation rate, exhibit high levels of polymorphism (Selkoe and Toonen 2006; Zhang and Hewitt 2003). Multiple alleles can be recovered per locus, allowing the detection of genetic structure among closely related populations and the study of contemporary demographic processes (Ellegren 2004; Schlötterer 2000; Zhang and Hewitt 2003).

The reliability of SSRs has been further enhanced by the emergence of next‐generation sequencing techniques, enabling the identification of countless loci in non‐model species at reduced costs (Curto et al. 2013; Guichoux et al. 2011). Furthermore, unlike traditional approaches using capillary electrophoresis, genotyping SSRs via high‐throughput amplicon sequencing considers both amplicon length and sequence information, thereby capturing all essential polymorphic features (Curto et al. 2019; De Barba et al. 2017; Tibihika et al. 2018). However, microsatellite markers also feature inherent constraints. For instance, mutations within the flanking regions are common and can impede primer annealing, resulting in an increased number of null alleles (Callen et al. 1993; Jarne and Lagoda 1996).

To compensate for these constraints and increase the robustness of a marker set, it is therefore advisable to include additional complementary markers, such as intronic sequences (Zhang and Hewitt 2003). Here, primers are typically designed to anneal to flanking exon regions and amplify across an intron, an approach termed exon‐primed intron‐crossing (EPIC) markers. Exons are well conserved and thus constitute stable primer annealing sites, minimizing the risk of null alleles. Introns, however, show high levels of genetic variation (Lessa 1992). Although frequently employed in inter‐species phylogenies (Creer 2007; Curto et al. 2012; Li et al. 2010), they are particularly effective for uncovering intraspecific genetic diversity (Lessa 1992; Palumbi and Baker 1994; Slade et al. 1993; Thomson et al. 2010). Exhibiting lower mutation rates compared to microsatellites (Li et al. 2002; Thomson et al. 2010), they are known to provide a realistic representation of population evolutionary history at deeper time frames (Edwards and Bensch 2009). The rapidly growing number of publicly available high‐quality genomes has strongly facilitated the applicability of EPIC markers. Using databases like NCBI (https://www.ncbi.nlm.nih.gov), it is possible to screen innumerable genomes for suitable loci and coordinate primer design with sequences of other taxa to increase the markers' reliability and taxonomic range (Backström et al. 2008; Curto et al. 2012; Li et al. 2010). Typically, EPIC markers are sequenced using capillary sequencing platforms (Lohse et al. 2011; White et al. 2015; Yao et al. 2013). However, like SSRs, their application can be streamlined by using high‐throughput amplicon sequencing.

Generally, genotyping by high‐throughput amplicon sequencing (GBAS) is easily standardized, clearly reproducible, and cost‐effective, making it ideal for long‐term population genetic research, such as genetic monitoring (Curto et al. 2019; De Barba et al. 2017; Tibihika et al. 2018). Genetic monitoring, the surveillance of population genetic parameters and intraspecific variation, has become an indispensable tool of modern conservation biology (Schwartz et al. 2007). Human‐induced habitat fragmentation, such as road construction and settlements, substantially contributes to the reduction of overall population size and the isolation of smaller sub‐populations. These changes result in increased inbreeding and reduced or even disrupted gene flow between fragmented sub‐populations, thereby decreasing genetic diversity (DiBattista 2008). Low genetic diversity within a population correlates with reduced fitness and consequently an elevated risk of extinction (Reed and Frankham 2003). Genetic monitoring makes these processes tangible, allowing for improved conservation measures. For example, it can highlight the need for increased protection of completely isolated sub‐populations, whose local extinction cannot be mitigated by migration from other populations (Levins 1969).

Amphibians are particularly affected by anthropogenic land transformation. Along with fungal pathogens and pollution, land degradation and urbanization are the major drivers of their global decline (Beebee and Griffiths 2005; Hamer and McDonnell 2008; Scheffers and Paszkowski 2012). Nonetheless, some amphibian species have shown an ability to cope with urban environments to some extent. For instance, the green toad ( *Bufotes viridis* ), whose primary habitats—steppes and wild river floodplains of Europe—suffer from extensive development and urbanization, is now frequently found in urban areas (Bogdan 2014; Konowalik et al. 2020; Mazgajska and Mazgajski 2020; Sistani et al. 2021; Vargová et al. 2023). However, these areas often provide only small, insular habitats where migration and thus gene flow may be restricted (Spießberger et al. 2023; Valkanova et al. 2009). It remains unclear whether these urban populations are genetically stable or suffer from low genetic diversity.

In this study, we developed a set of genetic markers for the green toad, *Bufotes viridis* . The SSR markers add to the 12 microsatellite loci developed by Vences et al. (2019), while the EPIC markers are, to our knowledge, the first identified for this species. We demonstrated the marker set's capability of elucidating population genetic parameters by genotyping green toad populations across urban and rural habitats. The marker set is applicable in GBAS and thus particularly suitable for long‐term population genetic research, such as genetic monitoring.

## 2 Materials and Methods

### 2.1 Sample Collection and DNA Isolation

**FIGURE 1:**
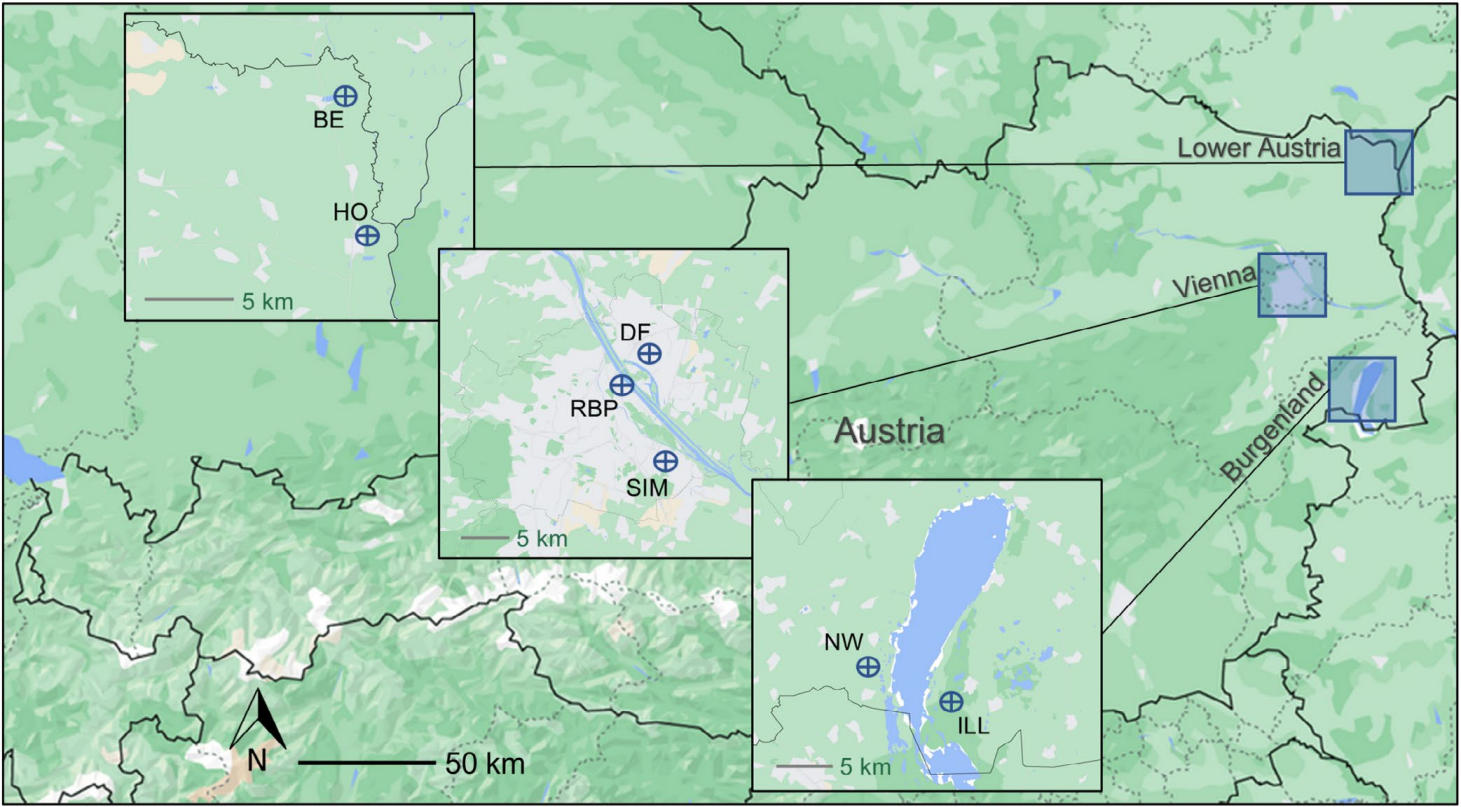
Sampling sites in the Austrian states of Burgenland and Lower Austria, as well as the federal capital, Vienna. BE: Bernhardsthal, DF: Donaufeld, HO: Hohenau, ILL: Illmitz, NW: Lake Neusiedl West, RBP: Rudolf‐Bednar‐Park, SIM: Simmering.

We utilized 62 green toad samples collected in 2023 and 2024. The samples comprised buccal swabs from living individuals as well as tissue from roadkills and tadpoles found dead. They originated from a total of seven sampling sites in the Austrian states of Burgenland and Lower Austria, as well as the federal capital, Vienna (Figure 1). The sampling sites in Burgenland—Lake Neusiedl West (four individuals) and Illmitz (four individuals)—and Lower Austria—Bernhardsthal (eight individuals) and Hohenau (five individuals)—were temporary or permanent natural water accumulations located in rural areas characterized by little urban development. In contrast, the sites in Vienna—Donaufeld (9 individuals), Rudolf‐Bednar‐Park (13 individuals), and Simmering (18 individuals)—were artificially created waterbodies (e.g., ponds and fountains) in urban environments, surrounded by settlements, roads, and other man‐made structures. The highest degree of urbanization was to be found in Rudolf‐Bednar‐Park. Here, a small set of shallow ornamental ponds is situated in a park within a heavily developed district close to the city center.

Except for transportation, we continuously stored both buccal swabs and tissue samples at −20°C. Given the green toad's classification as vulnerable, endangered, and critically endangered in Burgenland, Lower Austria, and Vienna, respectively (Gollmann 2007), we obtained sampling permits from the individual states. The permit numbers were “A4/NR.AB‐10090‐11‐2023” (Burgenland), “RU5‐BE‐1856/001‐2022” (Lower Austria), and “MA22‐230917‐2020” (Vienna).

For tissue lysis, we incubated each sample in 400 μL of lysis buffer (2% SDS, 2% PVP 40, 250 mM NaCl, 200 mM Tris–HCl, 5 mM EDTA at pH 8) containing 13 μL of Proteinase K (10 mg/mL) at 56°C and 350 rpm for approximately 3 h. Subsequently, we added 13 μL of RNase (10 mg/mL) and continued incubation at 37°C and 350 rpm for 15 min. For protein precipitation, we introduced 100 μL of cool 3 M KOAc and placed the samples on ice for another 20 min. Following stepwise centrifugation, we combined 300 μL of the clear lysate (supernatant) with 7.5 μL magnetic beads (MagSi‐DNA beads, MagnaMedics, Aachen, Germany) and 400 μL binding buffer in a 1.2 mL 96 well plate. We then extracted the magnetic beads along with the bound DNA using an inverted magnetic bead extraction device, VP 407‐AM‐N (V&P Scientific INC., San Diego, USA), washed them twice in 200 μL 80% EtOH, and ultimately eluted the DNA two times with 50 μL elution buffer (10 mM Tris–HCl at 65°C and pH 8.3).

For buccal swab samples, we performed cell lysis combining a clipped swab with 500 μL of lysis buffer and 16 μL of Proteinase K. After a 2.5‐h incubation at 56°C and 350 rpm, we transferred the swab into a spin column (NucleoSpin Filter, MACHEREY‐NAGEL, Düren, Germany) and centrifuged it stepwise to receive the entire amount of lysate. We mixed 450 μL of lysate with 675 μL of binding buffer and passed it through a silica membrane column (EconoSpin) via centrifugation. Next, we washed the membrane twice using 600 μL of 80% EtOH and eluted the DNA two times with 50 μL of elution buffer.

For both methods, we assessed the quality of the isolated DNA on 0.8% agarose gels.

### 2.2 SSR Marker Identification and Primer Design

We sent isolated high quality genomic DNA of a tissue sample from Illmitz, Burgenland, for library preparation and whole‐genome shotgun sequencing (Illumina MiSeq paired‐end 300 bp) to the Genomics Service Unit, Ludwig‐Maximilian's‐Universität München, Germany. We isolated microsatellite containing sequences as described in Curto et al. (2019). Here, we defined a minimum of 10 repeats for 2mers, 8 for 3mers, and 4 for 4/5mers as well as flanking regions longer than 20 base pairs on both sides of the motif. The outcome comprised a total of 10,381 SSR motif reads, of which 4683 were 2mers, 640 3mers, 4827 4mers, and 231 5mers.

Considering that motifs with shorter repeat unit lengths are more susceptible to *Taq* polymerase slippage (Ellegren 2004; Schlötterer and Tautz 1992; Shinde et al. 2003), we decided to exclusively feed sequences containing 4mer and 5mer motifs into Geneious v. 8.0.5 (https://www.geneious.com) for subsequent primer design. We designed primers utilizing the implemented tool Primer3 (Untergasser et al. 2012) specifying the following characteristics: lengths between 18 and 22 bp, an optimal melting temperature (Tm) of 55°C, a Tm difference of less than 5°C between forward and reverse primers, and target PCR product sizes between 425 and 470 bp. Additionally, we aimed for G/C contents between 40% and 60% and one to two G/C pairs at both the 5′ and 3′ ends to improve binding stability. We manually ascertained that the complete microsatellite repetition motif was located well within the first or last 250 bases of the resulting amplicon to allow merging of paired reads in 300 bp MiSeq as well as 250 bp NovaSeq 6000 runs. To reduce the likelihood of designing primers in duplicated loci, we mapped the FASTQ file of our genomic library against a list of all primers with Geneious' “Map to Reference” command and discarded any primer that had more than one read aligned to it. Also, we blasted (Altschul et al. 1990) each primer sequence against a publicly available green toad genome (accession number GCA_033119425.1) in the NCBI database (https://www.ncbi.nlm.nih.gov) to ensure the primers did not originate from contamination. Furthermore, this step served as an additional check for duplication, given the potentially incomplete genomic library. It also showed whether the primers aligned perfectly with sequences from at least two distinct individuals, increasing the likelihood of cross amplification. Eventually, we had a set of 48 primer pairs that met our requirements.

To enable Illumina sequencing, we elongated the 5′‐ends of the forward and reverse primers with a part of the Illumina adapters P5 (TCTTTCCCTACACGACGCTCTTCCGATCT) and P7 (CTGGAGTTCAGACGTGTGCTCTTCCGATCT), respectively. These adapter parts represent recognition sequences later required for a second PCR, which adds 8 bp of index information and the remaining parts of the Illumina adapters (P5: AATGATACGGCGACCACCGAGATCTACAC [Index] ACACTCTTTCCCTACACGACG; and P7:CAAGCAGAAGACGGCATACGAGAT [Index] GTGACTGGAGTTCAGACGTGT).

### 2.3 EPIC Marker Identification and Primer Design

In the NCBI database, we searched for the genome of the closely related common toad ( *Bufo bufo* ), deposited under the accession number GCA_905171765.1 (Streicher 2021). We selected the interface “View annotated genes” and filtered exclusively for protein‐coding genes. Next, we examined the genes in the “Gene Table” format, in which the lengths of the exons and introns that constitute the respective gene are displayed. We identified and extracted sequences of contiguous exon pairs that were sufficiently long for primer design (e.g., more than 20 bp) and flanked an intron shorter than 400 bp. This selection criteria ensured the eventual production of amplicons around 450 bp in length, compatible with sequencing on both MiSeq and NovaSeq platforms. Subsequently, we accessed the green toad genome (accession number GCA_033119425.1), selected “BLAST the reference genome” and “Somewhat similar sequences (blastn)” (Altschul et al. 1997), and input one of the two associated common toad exons. We extended the resulting green toad sequence to include the intron as well as both exons under “Sequence ID” and “Change Region shown” and extracted it. Eventually, we aligned the two common toad exons with our green toad sequence employing the “Map to Reference” command in Geneious. This allowed us to identify conserved regions for primer design, potentially increasing the success rate of subsequent PCR reactions.

**FIGURE 2:**
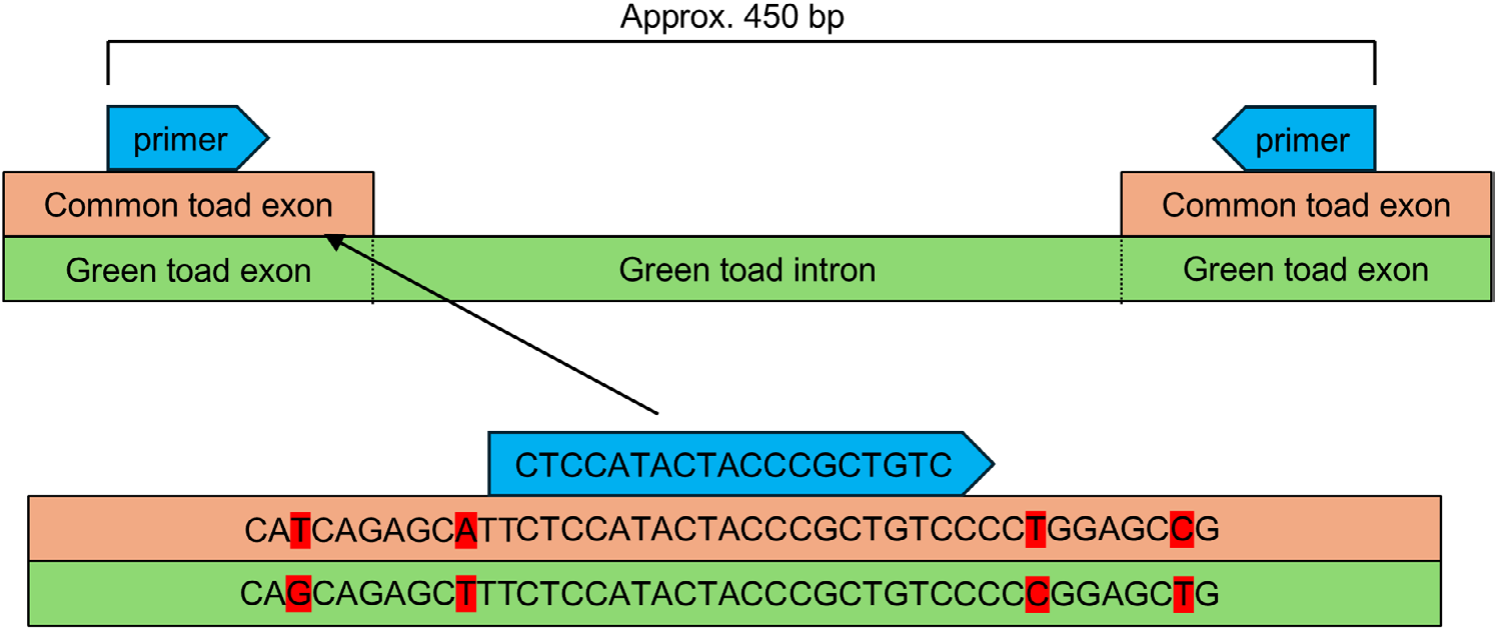
Schematic illustration of primer design for EPIC markers.

Using Geneious' Primer3, we positioned the forward primer on the exon at the 5′‐end of the alignment and the reverse primer on the opposite exon at the 3′‐end. We visually inspected whether the green toad and common toad sequences were identical at the primer site (Figure 2). In case of mismatches, we manually repositioned the primers. The primer characteristics and duplication checks were the same as for the SSR markers, resulting in another set of 48 primer pairs. Also, we adopted the preparation for Illumina sequencing.

### 2.4 Primer Testing

To examine the functionality of the developed primers, we individually tested them in singleplex PCRs. The final reaction volume was 10 μL, comprising 5 μL of Multiplex PCR Master Mix (QIAGEN), 2 μL of each primer (forward and reverse, 1 μM), and 1 μL of template DNA. For every primer pair, we performed three separate PCRs, using a distinct DNA isolate for each reaction. We selected isolates originating from different sampling sites, two from Vienna (Rudolf‐Bednar‐Park and Simmering) and another one from Burgenland (Illmitz), to assess the primers' transferability between populations. The PCRs followed this temperature profile: an initial denaturation step at 95°C for 15 min, followed by 35 cycles of denaturation (95°C for 30 s), annealing (55°C for 1 min), and extension (72°C for 1 min) as well as a final extension at 72°C for 10 min. Ultimately, we visualized the results on 1.5% agarose gels, yielding 48 and 45 positive amplifications at the target length (450 bp) for the EPIC and SSR markers, respectively.

### 2.5 PCR Multiplex

We grouped primer pairs working in the singleplex PCRs test into 4 EPIC mixes, each containing 11–13 primer pairs, and 4 SSR mixes, each with 11–12 primer pairs. To allocate primer pairs to the respective mixes, we utilized the software PrimerPooler (Brown et al. 2017). Additionally, we prepared two comprehensive mixes: one comprising all EPIC primer pairs and the other including the 45 functional SSR primer pairs. Each primer had a final concentration of 1 μM in the respective mix. We performed subsequent multiplex PCRs in a total volume of 10 μL containing 5 μL Multiplex PCR Master Mix (QIAGEN), 3 μL H_2_O, 1 μL primer mix, and 1 μL template DNA. For a subset of the samples, we utilized the Liquid Handling Station (Brand, Wertheim, Germany) to prepare the reactions of a total volume of 5 μL with the same reagent content ratio. Altogether, we used 61 DNA isolates, excluding the one used for creating the genomic library. The temperature profile for the multiplex PCRs was analogous to that of the singleplex PCRs, with the exception that we reduced the number of cycles to 30. PCRs with the eight primer mixes containing 11–13 primer pairs produced bands at the correct position (450 bp) on 1.5% agarose gels. In contrast, reactions using the two comprehensive mixes resulted only in primer dimers. Consequently, we discarded these mixes.

### 2.6 Purification, Index PCR, and Illumina Sequencing

For each sample and marker type, we combined PCR products from different primer mixes into single pools. We did the same for the singleplex PCR products. To remove primer dimers and unused primers, we purified each pool following a slightly modified Agencourt AMPure XP PCR Purification protocol. We started by adding 2.9 μL of AMPure XP beads (Beckman Coulter Inc., Brea, USA) to 4 μL of pooled PCR products and incubating the mixture at room temperature for 5 min. Subsequently, we captured bound DNA beads with a VP 407‐AM‐N inverted magnetic bead extraction device (V&P Scientific INC., San Diego, USA) and washed them twice in 150 μL of 80% ethanol for 45 s. Ultimately, we dried the beads at room temperature for another 5 min before eluting the bound DNA using 30 μL of elution buffer (10 mM Tris–HCl at 65°C and pH 8.3).

For the second PCR, mentioned in the “SSR marker identification and primer design” section, we selected a unique combination of forward and reverse indices for each PCR product pool, allowing reliable identification after the MiSeq run. The total reaction volume of the PCR was 10 μL, consisting of 5 μL QIAGEN Multiplex PCR Master Mix, 2 μL of each primer (1 μM), and 1 μL of the purified, pooled PCR products. The temperature profile included an initial denaturation step at 95°C for 15 min, 10 cycles of 95°C for 30 s, 58°C for 1 min, and 72°C for 1 min, followed by a final extension at 72°C for 5 min. As a result, the PCR products from the individual pools included, from the 5′ to 3′‐end, the following components: (1) Illumina P5 motif for flow cell hybridization, (2) 8‐bp index 1, (3) P5 sequencing primer, (4) specific forward primer, (5) target DNA for sequencing, (6) specific reverse primer, (7) P7 sequencing primer, (8) 8‐bp index 2, and (9) Illumina P7 motif for flow cell hybridization. Finally, we visualized the PCR products on a 1.8% agarose gel, combined all into a single pool, and sent them for 300 bp paired‐end sequencing (Illumina MiSeq) to the Genomics Service Unit at Ludwig‐Maximilian's‐Universität München, Germany.

### 2.7 Sequence Analysis and GBAS

We processed raw sequence data using custom Python and R scripts (github.com/mcurto/SSR‐GBS‐pipeline) as described in Curto et al. (2019). These scripts, initially developed for the processing of SSR markers only (Curto et al. 2019; Tibihika et al. 2018), were later adapted to include EPIC markers as well. They perform allele calling based on both allele length and sequence information, considering SNP variation within the alleles.

We visually inspected the two resulting co‐dominant allele matrices, one for SSR and one for EPIC markers, and removed the singleplex PCR primer test results as we did not require them for subsequent analyses. Instead, we used these results to trace whether potential marker failure was due to general non‐functionality or other issues such as primer dimer formation in the mixes or diverging primer annealing sites in certain populations. Next, we excluded samples yielding more than 30% missing data across all markers. Ultimately, we discarded markers that failed in more than 30% of the remaining samples. We selected the 30% threshold because it mirrored the results of more stringent thresholds in subsequent population genetic analyses, while retaining more samples and markers.

### 2.8 Population Genetic Analysis

After examining marker functionality, information content as well as their amplification success across all sampled populations, we used the data obtained for population genetic analyses. First, we excluded markers that were monomorphic across the entire dataset and thus uninformative. Additionally, we decided to exclude populations with fewer than five individuals to increase the robustness of the results, selecting this cutoff based on its effectiveness in previous studies with comparable sample sizes (Baptista et al. 2024, 2021). Next, we created a third matrix combining the SSR and EPIC marker co‐dominant allele matrices. We analyzed the three processed matrices separately using the cross‐platform Excel package GenAlEx v. 6.51b2 (Peakall and Smouse 2006) deriving several variability measures, in particular, the mean number of different alleles (*N*_a_), mean number of effective alleles (*N*_e_), mean observed heterozygosity (*H*_o_), and mean expected heterozygosity (*H*_e_). Additionally, we tested each marker for deviations from Hardy–Weinberg equilibrium (HWE), calculated Wright's Fixation Index or inbreeding coefficient (*F*_IS_), and explored patterns of differentiation among population pairs with pairwise *F*_ST_ estimates. Using the program FreeNA (Chapuis and Estoup 2007), we estimated the proportion of null alleles. For proportions above 0.2, we assumed the presence of null alleles.

Furthermore, we visualized genetic differences among the individuals in a discriminant analysis of principal components (DAPC) utilizing the adegenet package (Jombart 2008) in R v. 4.1.1 (Ihaka and Gentleman 1996). We retained four PCA and DA axes, consistent with the biologically informative value of K‐1 proposed by Thia (2023). Additionally, we randomly downsampled all populations with more than five individuals to five and repeated the analysis to assess whether unequal sample sizes distorted DAPC clustering distances.

Using STRUCTURE v. 2.3.4 (Hubisz et al. 2009), we investigated the individuals' genetic clustering. We defined the following parameters: the number of clusters (*K*) ranging from 2 to 6, 100,000 generations, a burn‐in period of 10,000, and 10 iterations. We estimated the optimal *K*‐value with delta *K* (∆*K*) using the Evanno method (Evanno et al. 2005) implemented in StructureSelector (https://lmme.ac.cn/StructureSelector). As the Evanno method can underestimate population structure (Janes et al. 2017), we carefully evaluated the suggested *K*‐values. Specifically, we visually inspected whether higher *K*‐values revealed additional, clearly delineated clusters or merely introduced noise. In case of the former, we focused more on the higher *K*‐value.

## 3 Results

### 3.1 Sequence Analysis and GBAS

GBAS initially produced a total of 12,525,989 paired‐end reads. Quality control, read merging, and demultiplexing reduced this number to 3,008,676, resulting in an average of 997 reads per sample and marker. The raw reads are available through the BioProject accession number PRJNA1218933. Excluding samples with more than 30% missing data across all markers narrowed our dataset to 46 individuals. Eventually, it comprised 6 individuals from Bernhardsthal, 5 from Hohenau, 3 from Illmitz, 2 from Lake Neusiedl West, 9 from Donaufeld, 5 from Rudolf‐Bednar‐Park, and 16 from Simmering. Discarding markers that failed in more than 30% of the remaining samples in the multiplex PCRs resulted in a set of 35 SSR and 46 EPIC markers. The raw and filtered co‐dominant allele matrices, following sample exclusion and marker discards, are provided in Appendix S1. Of the 12 markers discarded, two (Bv41_SSR and Bv48_SSR) had also failed to produce sequencing results in the singleplex PCRs (Tables A1 and A2), despite yielding bands at the correct position on agarose gels.

### 3.2 Population Genetic Analysis

The markers that turned out to be monomorphic across all investigated populations were: Bv12_SSR, Bv14_SSR, Bv20_SSR, Bv23_SSR, Bv40_SSR, Bv14_EPIC, Bv46_EPIC, and Bv48_EPIC. Subsequent population genetic analyses therefore do not include them. Furthermore, the populations of Illmitz and Lake Neusiedl West were represented by less than five individuals. Consequently, we did not consider these populations in subsequent analyses.

### 3.3 Analysis Using SSR Markers

Using only the SSR markers, we found that the mean number of different alleles (N_a_) ranged from 2.367 in rural Hohenau to 4.467 in urban Simmering. Similarly, the number of effective alleles (*N*_e_) showed the lowest average in Hohenau, at 2.008, and the highest in Simmering, at 2.707. Furthermore, N_a_ continuously exceeded *N*_e_. Observed heterozygosity (*H*_o_) and expected heterozygosity (*H*_e_) followed the same pattern, with *H*_o_ constantly higher than *H*_e_. Mean *H*_o_ varied between 0.444 in the urban Donaufeld population and 0.586 in rural Bernhardsthal, while mean *H*_e_ ranged from 0.405 in Hohenau to 0.520 in Simmering (Table 1a). None of the 30 investigated SSR markers deviated significantly from HWE across all populations. However, significant deviations within individual populations occurred in 13 markers (Table 1a). The *p*‐values are summarized in the supporting information alongside the associated *F*_IS_ values (Table S1). Additionally, none of the markers showed a proportion of null alleles above 0.2 in all populations, as revealed by FreeNA. Bv25_SSR and Bv4_SSR surpassed this threshold in Bernhardsthal, Donaufeld, and Simmering. However, excluding them from the analysis produced identical results, so we retained them in the marker set. The lowest pairwise *F*_ST_ value occurred between rural Hohenau and urban Rudolf‐Bednar‐Park at 0.050. Donaufeld and Simmering, both urban, were just above this at 0.064. We observed the highest pairwise *F*_ST_ value between the rural populations from Bernhardsthal and Hohenau (0.148). Only slightly below was the value between Donaufeld and Hohenau (0.144; Table 2a).

**TABLE 1:** Variability measures (mean ± SE), including mean number of individuals per locus with genotype data (*N*), mean number of different alleles (*N*_a_), mean number of effective alleles (*N*_e_), mean observed heterozygosity (*H*_o_), and mean expected heterozygosity (*H*_e_), for each population with five or more individuals.

	Population	*N*	*N*_a_	*N*_e_	*H*_o_	*H*_e_	HWE dev.
(a)	Bernhardsthal	5.867 ± 0.104	2.933 ± 0.283	2.374 ± 0.251	0.586 ± 0.065	0.473 ± 0.040	4
	Donaufeld	8.967 ± 0.033	2.967 ± 0.337	2.187 ± 0.245	0.444 ± 0.056	0.426 ± 0.044	9
	Hohenau	4.967 ± 0.033	2.367 ± 0.206	2.008 ± 0.157	0.505 ± 0.064	0.405 ± 0.046	3
	Rudolf‐Bednar‐Park	4.933 ± 0.046	2.600 ± 0.274	2.159 ± 0.212	0.507 ± 0.066	0.418 ± 0.048	1
	Simmering	15.867 ± 0.079	4.467 ± 0.531	2.707 ± 0.274	0.527 ± 0.056	0.520 ± 0.043	10
(b)	Bernhardsthal	5.953 ± 0.032	2.279 ± 0.157	1.711 ± 0.090	0.436 ± 0.046	0.347 ± 0.033	2
	Donaufeld	8.884 ± 0.095	2.279 ± 0.126	1.694 ± 0.094	0.359 ± 0.039	0.340 ± 0.033	3
	Hohenau	4.140 ± 0.222	1.884 ± 0.174	1.530 ± 0.129	0.269 ± 0.040	0.269 ± 0.039	1
	Rudolf‐Bednar‐Park	4.767 ± 0.065	1.837 ± 0.105	1.559 ± 0.080	0.302 ± 0.047	0.286 ± 0.035	6
	Simmering	15.907 ± 0.065	2.907 ± 0.207	2.022 ± 0.151	0.411 ± 0.041	0.407 ± 0.037	6
(c)	Bernhardsthal	5.918 ± 0.047	2.548 ± 0.152	1.984 ± 0.121	0.497 ± 0.039	0.399 ± 0.027	
	Donaufeld	8.918 ± 0.058	2.562 ± 0.161	1.897 ± 0.117	0.394 ± 0.033	0.375 ± 0.027	
	Hohenau	4.479 ± 0.139	2.082 ± 0.135	1.726 ± 0.103	0.366 ± 0.037	0.325 ± 0.031	
	Rudolf‐Bednar‐Park	4.836 ± 0.044	2.151 ± 0.135	1.805 ± 0.104	0.386 ± 0.040	0.340 ± 0.029	
	Simmering	15.890 ± 0.050	3.548 ± 0.264	2.303 ± 0.148	0.459 ± 0.034	0.453 ± 0.029	

*Note:* The table also includes the number of markers deviating from HWE (HWE dev.). (a) Using only the SSR markers, (b) using only the EPIC markers, (c) using the SSR and EPIC markers combined.

**TABLE 2:** Pairwise *F*_ST_ values for populations with five or more individuals.

	Nei genetic distance			Pairwise population *F*_ST_ values		
	Bernhardsthal	Donaufeld	Hohenau	Rudolf‐Bednar Park	Simmering	
(a)	0.000					Bernhardsthal
	0.105	0.000				Donaufeld
	0.148	0.144	0.000			Hohenau
	0.129	0.134	0.050	0.000		Rudolf‐Bednar‐Park
	0.074	0.064	0.113	0.088	0.000	Simmering
(b)	0.000					Bernhardsthal
	0.106	0.000				Donaufeld
	0.132	0.172	0.000			Hohenau
	0.124	0.133	0.189	0.000		Rudolf‐Bednar‐Park
	0.076	0.105	0.138	0.101	0.000	Simmering
(c)	0.000					Bernhardsthal
	0.106	0.000				Donaufeld
	0.140	0.160	0.000			Hohenau
	0.126	0.133	0.128	0.000		Rudolf‐Bednar‐Park
	0.076	0.088	0.127	0.096	0.000	Simmering

*Note:* (a) Using only the SSR markers, (b) using only the EPIC markers, (c) using the SSR and EPIC markers combined.

**FIGURE 3:**
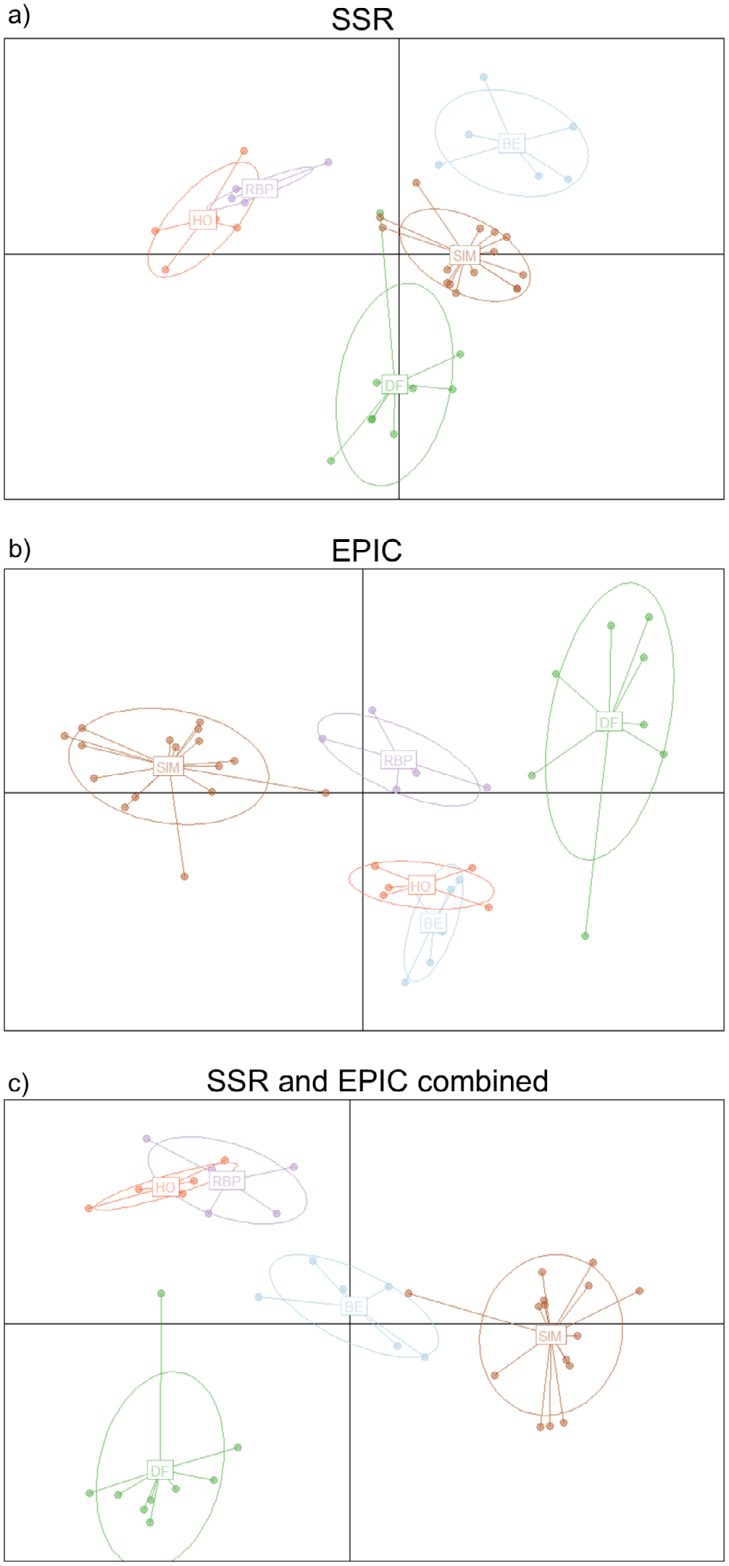
Genetic differences among all green toad populations, visualized in a discriminant analysis of principal components (DAPC). (a) Using only the SSR markers, (b) using only the EPIC markers, and (c) using the SSR and EPIC markers combined. BE: Bernhardsthal, DF: Donaufeld, HO: Hohenau, RBP: Rudolf‐Bednar‐Park, SIM: Simmering.

The DAPC revealed four major clusters. The rural Bernhardsthal population as well as the urban populations from Donaufeld and Simmering formed distinct groups, except for one Donaufeld outlier that overlapped with Simmering (Figure 3a). Despite the considerable geographic distance (Figure 1) and the barriers posed by the urban environment, the inner‐city Rudolf‐Bednar‐Park population and the one from rural Hohenau represented a single cluster (Figure 3a). The DAPC using the downsampled dataset with five individuals per population produced identical clustering patterns (Figure S1a).

**FIGURE 4:**
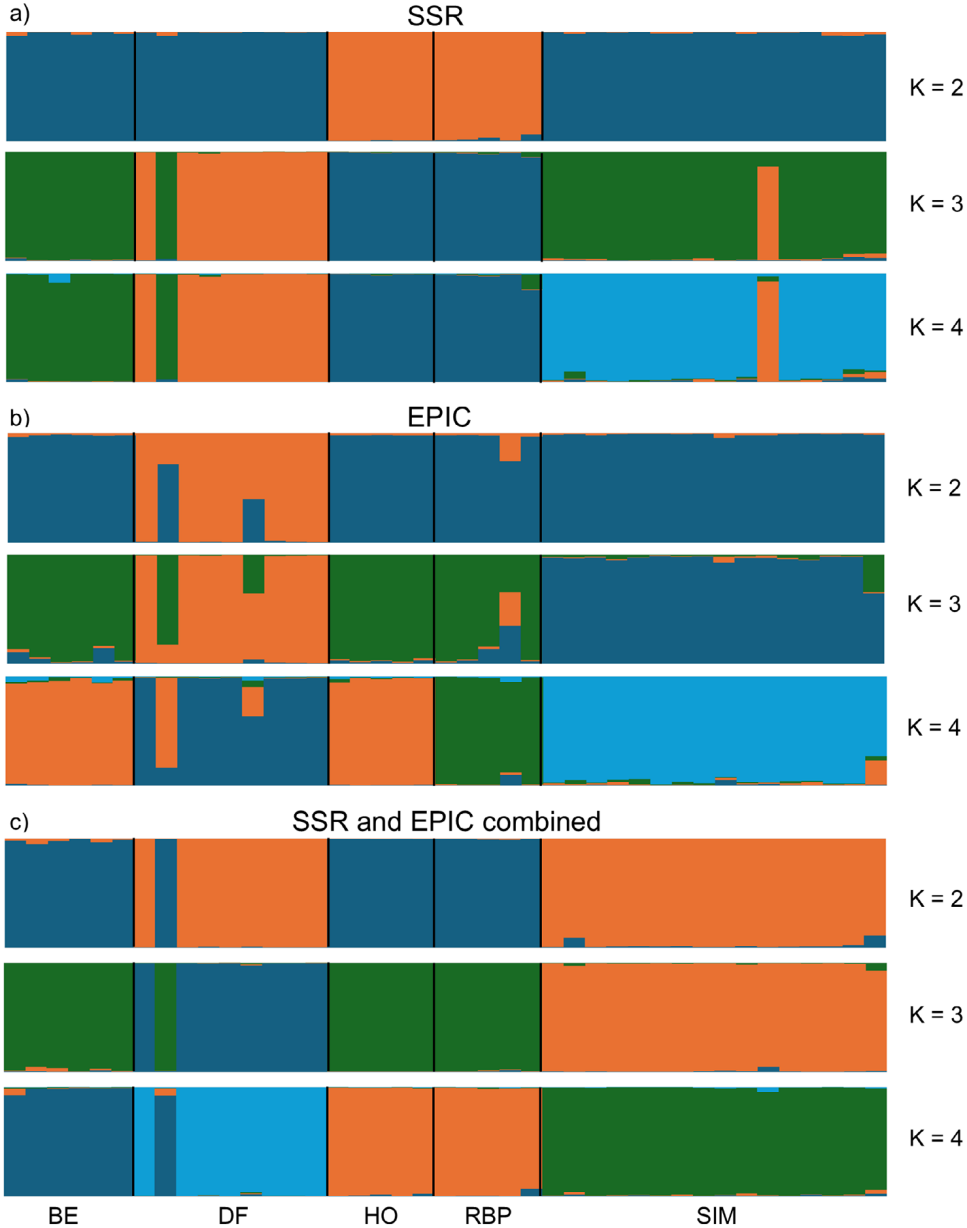
Genetic clustering of green toad individuals from all populations, derived from STRUCTURE v. 2.3.4. (a) Using only the SSR markers, (b) using only the EPIC markers, and (c) using the SSR and EPIC markers combined. BE: Bernhardsthal, DF: Donaufeld, HO: Hohenau, RBP: Rudolf‐Bednar‐Park, SIM: Simmering.

Delta *K* (∆*K*) peaked at *K* = 4. Moreover, higher *K*‐values did not reveal additional clustering patterns but instead introduced noise through over‐fragmentation within populations and spurious admixture. Thus, we considered *K* = 4 to be optimal. The STRUCTURE results aligned with the DAPC. For instance, all populations were individually separated, except for urban Rudolf‐Bednar‐Park and rural Hohenau, which were grouped together. STRUCTRE also identified the outlier of the urban Donaufeld population but assigned it to rural Bernhardsthal rather than urban Simmering. Moreover, the proximity of Simmering and Donaufeld was reflected by one Simmering individual more accurately matching Donaufeld (Figure 4a).

### 3.4 Analysis Using EPIC Markers

Considering only the EPIC markers, we observed the lowest mean number of different alleles, 2.367, in urban Rudolf‐Bednar‐Park, and the highest, 4.467, in urban Simmering. The mean number of effective alleles (*N*_e_) ranged from 1.530 in rural Hohenau to 2.022 in Simmering and remained constantly lower than *N*_a_. Observed heterozygosity (*H*_o_) exhibited average values between 0.269 in Hohenau and 0.436 in Bernhardsthal, which is also rural. Except for Hohenau, expected heterozygosity (*H*_e_) was consistently lower than *H*_o_ with average values between 0.269 (Hohenau) and 0.407 (Simmering; Table 1b). None of the investigated EPIC markers deviated significantly from HWE across all populations. Significant deviations in individual populations, however, occurred in 11 of the 43 tested markers (Table 1b). The *p*‐values as well as the associated *F*_IS_ values are shown in Table S1. As with the SSR markers, none of the EPIC markers had a proportion of null alleles above 0.2 across all populations, according to FreeNA. Bv4_EPIC exceeded this threshold in two populations, Rudolf‐Bednar‐Park and Simmering, showing the highest proportion of null alleles. We found the lowest pairwise *F*_ST_ value between rural Bernhardsthal and urban Simmering, at 0.076, while urban Rudolf‐Bednar‐Park and Simmering had the second lowest value, at 0.101. In contrast, the highest values occurred between rural Hohenau and Rudolf‐Bednar‐Park (0.189) as well as Hohenau and urban Donaufeld (0.172; Table 2b).

The DAPC also featured four major clusters (Figure 3b). In this analysis, however, rural Hohenau did not group with urban Rudolf‐Bednar‐Park, but with the geographically adjacent population from Bernhardsthal (Figure 1). The urban populations from Simmering, Rudolf‐Bednar‐Park, and Donaufeld formed clearly separable groups. Donaufeld had a prominent outlier again, which was located near the cluster comprising the rural populations Hohenau and Bernhardsthal (Figure 3b). The downsampled DAPC using five individuals per population recovered the same groups (Figure S1b).

Delta *K* (∆*K*) peaked at *K* = 3. However, *K* = 4 revealed an additional, clearly delineated cluster consistent with our sampling sites without adding noise. In comparison, *K* = 5 contributed only noise. Consequently, we determined *K* = 4 to be optimal. As with the SSR markers, the STRUCTURE results aligned accurately with the DAPC. Specifically, the rural populations from Hohenau and Bernhardsthal grouped together, whereas the urban populations from Simmering, Rudolf‐Bednar‐Park, and Donaufeld each formed distinct clusters. Additionally, STRUCTURE identified the outlier within the Donaufeld population, assigning it primarily to Bernhardsthal (Figure 4b).

### 3.5 Integrated Analysis of SSR and EPIC Markers

Using SSR and EPIC markers in a combined approach, the mean number of different alleles (*N*_a_) ranged from 2.082 in rural Hohenau to 3.548 in urban Simmering. The number of effective alleles (*N*_e_), with mean values between 1.726 (Hohenau) and 2.303 (Simmering), was consistently lower than *N*_a_. Similarly, mean expected heterozygosity (*H*_e_) constantly fell short of mean observed heterozygosity (*H*_o_). *H*_e_ values ranged from 0.325 (Hohenau) to 0.453 (Simmering). *H*_o_ values varied between 0.366 in Hohenau and 0.497 in neighboring Bernhardsthal (Table 1). Pairwise *F*_ST_ values were lowest among the populations from rural Bernhardsthal and urban Simmering (0.076), followed by urban Donaufeld and Simmering (0.088). Highest values occurred when comparing Donaufeld and rural Hohenau (0.160), as well as Bernhardsthal and Hohenau (0.140; Table 2c).

Like the preceding DAPC analyses, the one based on both the SSR and EPIC markers showed four major clusters. Rural Hohenau did not cluster with neighboring Bernhardsthal but rather with the urban population from Rudolf‐Bednar‐Park, consistent with the pattern observed in the SSR‐only DAPC. The remaining urban populations, Simmering and Donaufeld, formed separate clusters, each with outliers located near Bernhardsthal (Figure 3c). The downsampled DAPC with five individuals per population produced the same clustering pattern, although the overlap between Hohenau and Rudolf‐Bednar‐Park was less pronounced (Figure S1c).

The highest delta *K* (∆*K*) occurred at *K* = 4. Furthermore, higher *K*‐values merely introduced noise. Thus, we considered *K* = 4 to be optimal. As with the DAPC, STRUCTURE assigned all populations, except rural Hohenau and urban Rudolf‐Bednar‐Park, to distinct groups. One individual from urban Donaufeld, however, primarily grouped with rural Bernhardsthal (Figure 4c).

## 4 Discussion

### 4.1 Implementing the GBAS Framework to EPIC Versus SSR Markers

Here, we present a set of 35 SSR and 46 EPIC markers for population genetic analysis in the green toad, *Bufotes viridis* . We propose their use in GBAS that can produce cheap genotyping data for large sample sizes in a reproducible way (Curto et al. 2019; Tibihika et al. 2018), enabling future studies to directly build on and expand our initial dataset. Potentially, this can facilitate the establishment of broader genetic monitoring, contributing to a comprehensive conservation strategy for the species and complementing ongoing green toad population monitoring in Austria and beyond (Burgstaller et al. 2024; Landler et al. 2023; Sistani et al. 2021; Staufer et al. 2023).

Of the 48 SSR markers initially developed, we discarded 13 due to failures in either singleplex or multiplex PCR. These results align with the literature and were anticipated as null allele occurrences due to mutations at primer annealing sites are a well‐known limitation of SSRs (Callen et al. 1993; Jarne and Lagoda 1996). Nonetheless, our SSR markers performed relatively well, exhibiting equal or lower failure rates compared to previous, analogous studies, despite our use of a more stringent threshold, retaining only markers that failed in fewer than 30% of samples (Baptista et al. 2024, 2021; Curto et al. 2019; Tibihika et al. 2018). This improved performance likely results from an additional quality control step not implemented in previous studies, where we compared primer sequences with the green toad genome in NCBI. This step served as an extra check for duplications and confirmed that the primers did not originate from contamination. Importantly, it also allowed us to retain only primers that aligned perfectly with sequences from at least two distinct individuals, increasing the probability of matching other individuals. The populations with the highest relative numbers of individuals exceeding the 30% missing data threshold for the SSR markers were Rudolf‐Bednar‐Park (8 out of 13) and Lake Neusiedl West (2 out of 4). With over 90%, the two Lake Neusiedl West individuals had a disproportionate amount of missing data, indicating either poor DNA quality or laboratory error rather than marker failure. The same applies to two Rudolf‐Bednar‐Park individuals, which showed 71% and 91% missing data, respectively. Additionally, four of the Rudolf‐Bednar‐Park individuals excluded merely exhibited missing data between 40% and 50%, narrowly exceeding our threshold (Appendix S1). Notably, these individuals would have met the criteria for inclusion in comparable studies (Baptista et al. 2024, 2021; Sinigaglia et al. 2024), suggesting that their exclusion reflects the stringency of our filtering rather than a limitation in marker performance.

The EPIC markers in this study performed exceptionally well. Of the 48 markers developed, all amplified successfully in singleplex tests, with only two failing in the multiplex setup. Low numbers of null alleles are a well‐reported and valuable feature of EPIC markers, largely due to the conserved nature of exons as stable primer annealing sites (Lessa 1992; Zhang and Hewitt 2003). Nonetheless, a considerably higher failure rate is typically associated with newly developed EPIC markers (Silva et al. 2017; Tay et al. 2008; White et al. 2015). Our study thus provides a particularly reliable set of EPIC markers for population genetic analyses in *Bufotes viridis* . The one population with the highest relative number of individuals exceeding the 30% missing data threshold was Lake Neusiedl West (two out of four). However, as with the SSR markers, this is more likely attributable to poor DNA quality, as indicated by a disproportionately high amount of missing data across all EPIC markers (81% and 94%). Notably, as we designed primers to also align with sequences of the common toad, *Bufo bufo* , the markers may be applicable in population studies of this species as well. Whether this is the case, however, remains to be investigated.

### 4.2 Population Genetics of *Bufotes viridis*

While the small and uneven sample sizes across populations pose limitations and should therefore be interpreted cautiously, our results suggest several emerging trends. For instance, allelic diversity does not appear to be generally lower in urban populations of *Bufotes viridis* . Neither the number of different alleles (*N*_a_) nor the number of effective alleles (*N*_e_) was continuously lowest in the urban populations. Regardless of the marker system used, the lowest values for *N*_a_ and *N*_e_ occurred in either rural Hohenau or urban Rudolf‐Bednar‐Park. The highest values constantly emerged in urban Simmering, followed by either rural Bernhardsthal or the urban population from Donaufeld, which showed largely similar values. Moreover, there was no clear differentiation between rural and urban populations regarding values for observed heterozygosity (*H*_o_). A recent publication by Vargová et al. (2023), employing traditional length‐based microsatellite genotyping in *B. viridis* , also did not show consistently reduced diversity in urban populations. In that study, already one of the three urban populations investigated did not differ from rural populations in the number of different alleles and observed heterozygosity (Vargová et al. 2023). In contrast, earlier studies on *Bufo bufo* , *Rana temporaria* , and *Pelophylax ridibundus* constantly showed lower *N*_a_ values and decreased *H*_o_ in urban populations compared to rural ones (Hitchings and Beebee 1998, 1997; Mikulíček and Pišút 2012). This suggests that the impact of urbanization and fragmentation on genetic diversity may vary among different anuran amphibian species, with *B. viridis* potentially showing greater resilience.

Furthermore, *H*_o_ consistently exceeded *H*_e_ in our results, except in the Hohenau population, where *H*_o_ and *H*_e_ were equal when considering only EPIC markers. Similarly, Vargová et al. (2023) reported higher *H*_o_ than *H*_e_ across both rural and urban populations of *Bufotes viridis* (Vargová et al. 2023). These findings collectively indicate that even in urban areas this species may maintain a sufficiently large effective population size to avoid inbreeding, further supporting the idea of resilience to such environments. *Pelophylax ridibundus* , for example, showed a deficit of heterozygotes in half of the urban populations. The authors of this study, however, stated that this might also be caused by the presence of null alleles (Mikulíček and Pišút 2012), hindering a comparison between the species.

Null alleles do not appear to pose a problem in our results. Among the 24 markers that deviated significantly from HWE in some populations, only two, Bv25_SSR and Bv4_SSR (both SSR markers), were likely affected by null alleles. While their highly positive associated *F*_IS_ values could also indicate inbreeding (Wright 1965), analysis with FreeNA attributed these deviations primarily to the presence of null alleles. Using EPIC markers, none of the significant deviations from HWE within individual populations originated from null alleles, further highlighting their value as a complement to SSR markers in overcoming such limitations.

Pairwise *F*_ST_ values did not reveal lower genetic differentiation among rural populations. Regardless of the marker system used, the neighboring rural populations from Bernhardsthal and Hohenau did not show lower pairwise *F*_ST_ values compared to those observed between rural and urban populations or between urban populations. This result contrasts with expectations of increased genetic differentiation in urban populations, as barriers such as roads and buildings typically limit connectivity (Johnson and Munshi‐South 2017). Indeed, higher genetic differentiation between urban populations of anuran amphibians has been reported in several studies (Hitchings and Beebee 1998, 1997; Mikulíček and Pišút 2012; Wei et al. 2021), including the green toad (Vargová et al. 2023). However, these studies benefited from considerably larger sample sizes, likely providing more robust estimates of genetic differentiation. The small and uneven sample sizes in our study may have biased the pairwise *F*_ST_ values, which is a known limitation of this method (Meirmans and Hedrick 2011).

Unlike pairwise *F*_ST_ calculations, DAPC and STRUCTURE do not rely on predefined population assignments (Weir and Cockerham 1984; Wright 1949). Instead, they analyze genetic data at the individual‐level without requiring prior assumptions about group memberships (Hubisz et al. 2009; Jombart et al. 2010; Pritchard et al. 2000), usually making these methods more robust to small and uneven sample sizes. This robustness is also supported by the congruency between our original DAPC and the version based on the downsampled dataset with five individuals per population. Furthermore, the consistency between our DAPC and STRUCTURE results enhances confidence in their reliability. Our DAPC based solely on SSR markers grouped each population into distinct clusters, except for rural Hohenau and urban Rudolf‐Bednar‐Park, which clustered together. STRUCTURE mirrored this pattern by assigning these two populations to a single group while separating all others individually. In contrast, Vargová et al. (2023) found all three rural populations and one urban population grouped together in both their principal component analysis (PCoA) and STRUCTURE results (Vargová et al. 2023). The more pronounced genetic structuring observed in our study is likely due to the ability of the GBAS procedure to detect finer‐scale genetic patterns than traditional length‐based microsatellite genotyping, as demonstrated in previous comparative studies (Curto et al. 2019; Tibihika et al. 2018). The clustering of rural Hohenau with Rudolf‐Bednar‐Park, the most isolated urban population in our study and located approximately 50 km apart, is most probably explained by genetic drift. Microsatellites, due to their high mutation rates, are particularly susceptible to genetic drift (Nauta and Weissing 1996). By increasing isolation and reducing gene flow, urbanization aggravates this effect, distorting patterns of population genetic structure (Johnson and Munshi‐South 2017; Miles et al. 2019). In our DAPC and STRUCTURE analysis using only EPIC markers, all three urban populations formed distinct clusters, while the rural populations from Bernhardsthal and Hohenau grouped together. Ecologically, this pattern appears more plausible than that derived from microsatellites, as the two rural sites are separated by merely 5 km, a distance readily traversed by the highly mobile green toad (Blab 1991; Indermaur et al. 2009; Stöck et al. 2008). Moreover, the largely undeveloped surrounding environment poses few barriers to gene flow. EPIC markers are less variable than microsatellites (Thomson et al. 2010) and, consequently, less affected by random drift‐effects. Therefore, they may provide ecologically more meaningful results in environments where genetic drift is likely to distort population genetic structure. Ultimately, the DAPC and STRUCTURE results derived from the combined SSR and EPIC markers were largely congruent with those based on the SSR markers only. The dominance of the SSR markers in these analyses is likely attributable to their higher allele numbers, a consequence of their inherently high polymorphism. This dominance was slightly reduced in the downsampled DAPC, as indicated by the weaker overlap between Hohenau and Rudolf‐Bednar‐Park.

## 5 Conclusion

Our findings collectively suggest that the green toad ( *Bufotes viridis* ) exhibits a degree of resilience to urban environments. We demonstrated that urban green toad populations in Vienna do not necessarily have reduced genetic diversity compared to rural populations. Whether this pattern holds true in other urban settings remains to be investigated. Furthermore, we highlighted the value of EPIC markers as a complement to SSR markers. In addition to having fewer null alleles, EPIC markers provide an effective buffer against random drift, which can otherwise distort patterns of population structure and lead to false conclusions. Therefore, we recommend combining EPIC and SSR markers, particularly in studies conducted in environments prone to genetic drift, such as urban areas.

## Author Contributions

**Vincent Kendlbacher:** conceptualization (equal), data curation (lead), formal analysis (lead), investigation (lead), methodology (equal), software (equal), visualization (lead), writing – original draft (lead), writing – review and editing (lead). **Yoko Philipina Krenn:** conceptualization (equal), data curation (supporting), funding acquisition (equal), investigation (supporting), project administration (equal), visualization (supporting), writing – review and editing (equal). **Thapasya Vijayan:** data curation (supporting), formal analysis (supporting), methodology (supporting), software (supporting), writing – review and editing (equal). **Christina Rupprecht:** software (supporting), writing – review and editing (equal). **Magdalena Spießberger:** data curation (supporting). **Manuel Curto:** methodology (supporting), software (supporting), writing – review and editing (equal). **Lukas Landler:** conceptualization (supporting), supervision (supporting), writing – review and editing (equal). **Harald Meimberg:** conceptualization (lead), funding acquisition (lead), project administration (lead), resources (lead), supervision (lead), writing – review and editing (equal).

## Conflicts of Interest

The authors declare no conflicts of interest.

## Supporting information


**Figure S1.** Discriminant analysis of principal components (DAPC) showing all populations downsampled to five individuals. (a) Using only the SSR markers, (b) using only the EPIC markers, (c) using the SSR and EPIC markers combined. BE: Bernhardsthal, DF: Donaufeld, HO: Hohenau, RBP: Rudolf‐Bednar‐Park, SIM: Simmering.

**Table S1.** Test for deviations from Hardy–Weinberg equilibrium (HWE) and values for Wright’s Fixation Index (*F*_IS_) per marker, for populations with five or more individuals.

Appendix S1.

## Data Availability

All relevant data are within the manuscript and the publicly available Sequence Read Archive (SRA) under the BioProject accession number PRJNA1218933.

## Appendix A

**TABLE A1:** Final set of 35 SSR primer pairs, including the mix in which we used them for multiplex PCR (Note), the repetition motifs (Motif), the primer sequences (forward and reverse), and the allele length ranges (amplicon length variation). The table also includes the 13 SSR primers that failed in either the singleplex or multiplex PCRs, as specified in the 'Note' column.

Primer name	Note	Motif	Forward	Reverse	Amplicon length variation
Bv6_SSR	Mix 1	TCTA	GTTTGTGCATTCAATTGGC	GTGCTGTGGTTACGTTTTAT	434–482
Bv14_SSR	Mix 1	TTAT	GAACCTCCACTGACCAATC	GTTTTCTTCATCCCTCCTCC	448
Bv24_SSR	Mix 1	AAACA	GAGCTAGCTGACTACAATGG	GTCAGACCATGGGAAAAGAG	454
Bv30_SSR	Mix 1	GGAC	GGGAGACTGAGGAGACAG	GTTACAGGAAGAAACCGAAC	437–444
Bv35_SSR	Mix 1	GAAA	CCTGGGAGTTCAAAGTAAGG	GGACTAGACCTGGAGCTC	437
Bv39_SSR	Mix 1	GATA	GCAAGTACAATGGGAAAGC	AGTCTGAAGGGCTACTAGAG	438–482
Bv40_SSR	Mix 1	TTAT	CAATTGCCTCCTTTTCATGC	GACATATGGCCACAAAAACC	442
Bv47_SSR	Mix 1	CCAGA	CTCACTGCATAGCTCTTGC	CAATACTGGCAAGCGAGG	432
Bv9_SSR	Mix 2	TCTA	CGTGAGTTCTGGTGCATT	GTCCACTAACCACTGTAACC	440–464
Bv13_SSR	Mix 2	CATA	GTAGTGTGAGGTTCTGCTG	TTCGCTTTCCTGTTGATGTT	449
Bv17_SSR	Mix 2	ATGA	GTGACCCAACGTGCATAG	CAACCAAGCCACCATTCA	446–450
Bv20_SSR	Mix 2	AAAT	CAGTAGCAAAGGACGTATGT	GCATGGCAAACCGTTTCT	446
Bv23_SSR	Mix 2	AAAAT	GCACTCCATAATTCTGGTCC	GTGATAAGATCAGCCATTTGA	443
Bv33_SSR	Mix 2	GATA	CTTCATCGCCATAGTAACAC	GAGTCTCCTCCCCTTATCTC	430–503
Bv34_SSR	Mix 2	TTAT	GTCATCTCTACCAACATGTCG	TCCTTACTCAGTCTTCTGGC	439
Bv36_SSR	Mix 2	TATC	CAGGTGTATGCTACCAAGAG	GCGTCCCTATCTCTATTTCC	434–463
Bv45_SSR	Mix 2	TATC	GAGCAGGCATGTCTTCAG	CAGTGTTTGCCCCTGTAC	419–431
Bv1_SSR	Mix 3	AATA	CCGTGAACTGGTAGCATTG	CATTACCAGGCTCACAAAC	367–447
Bv5_SSR	Mix 3	AAAT	GGAATTTTGCCCCCGTTA	GATAGCTGCATAGGACATGA	442–443
Bv12_SSR	Mix 3	AAAT	TCTAGACTGTCTGCCATAGG	GTGAGTGATGGCTAACTAAG	451
Bv16_SSR	Mix 3	AGAT	CGTAGAGACTCCAATTACAAG	CTGAATAAAGCAGGTCCACC	405–450
Bv19_SSR	Mix 3	TATT	CAGTGGCCCGACATTTTAA	AACACAGGAAAACACGAAAC	444
Bv21_SSR	Mix 3	AAAC	GAGACTAACAGACACCCTTTA	CTGAGCAGGTGAAAAATATGTC	404–446
Bv32_SSR	Mix 3	TAGA	GACATGGACCATATAAGATGAC	CGCTTTCATCCTCCTGAC	342–426
Bv37_SSR	Mix 3	TCTA	GTACAAAGCTGCACAGATCC	GTGTCTGTGCACAAGTACA	464–492
Bv4_SSR	Mix 4	GAAA	GCTGCCTCCAGAGTTTTA	TTCGGCTTTGCTAATGTCTT	366–454
Bv15_SSR	Mix 4	TATC	GAAATCGCAGAGTGCTACG	GCTTTGTGTTGTGCTCTGA	293–421
Bv18_SSR	Mix 4	TATT	CAGGATCCATCTCTTGCAAC	CAACTGGGTGGAATGTTGTT	459–460
Bv22_SSR	Mix 4	TAGA	TAATAGTCAGTGGGTTTCTGC	GGAGTTCAGCTGACCTTTC	429–485
Bv25_SSR	Mix 4	AAGA	GCACATAGGATCCCAAAACC	TGTTTGGATTCATGTCTCTTG	422–437
Bv29_SSR	Mix 4	TCTA	CTACATTGTCGGGTTGGTG	CCTATACGGAAAGTAGACTACA	445
Bv31_SSR	Mix 4	TCTA	GCTCTCATATTTTGGAGGTTC	GGCCTTACCTTTGATACCTG	463
Bv43_SSR	Mix 4	AAAT	CATAAACAGTGAATGGAGGG	CAGCGTTCATAGTTTGGTAT	455–463
Bv44_SSR	Mix 4	ACAT	GCATGAAGGACAGACAGAC	CAAGCAGAGGGGAGTACAG	437–443
Bv46_SSR	Mix 4	TAAAA	CAGTGACTCCATCAGCTAC	GTTCACTGCCAGCAAAATG	436
Bv10_SSR	Failed in multiplex PCR	GATA	GAGGGAGGGCAACTTTTG	ATATGGGTCCCCTGTATGTG	—
Bv11_SSR	Failed in multiplex PCR	TTTA	CGGTGTGCTAGTCATTTATGA	CGAATGAAAGGAGATGTGGG	—
Bv2_SSR	Failed in multiplex PCR	AGAT	CAATCCAAGTTTACTCCAAGG	CCACTCCCTCTTCTCACTC	—
Bv26_SSR	Failed in multiplex PCR	TAGA	CCAAAGTGGAAAATCCTCTAG	CCTCTGTCTAAACTTTACACAC	—
Bv27_SSR	Failed in multiplex PCR	TTAT	GTAAATGTTTCGGGTTCGG	CTGCATATTCGGCAAACC	—
Bv3_SSR	Failed in multiplex PCR	GATA	GTCCAGTTGTATGCACGAA	ACCTTAACAACGTTTTGGAA	—
Bv42_SSR	Failed in multiplex PCR	TTGT	CTCTATGTGGATTTGATCAGG	TGTAAGACAGTGGAAGACAG	—
Bv7_SSR	Failed in multiplex PCR	TATC	GGCCCTCACTTATGATTTAGG	GCGCTCTTACTCCATGTG	—
Bv8_SSR	Failed in singleplex PCR	ATTT	TTAGTATGGGCAGCGAAAAG	AATCTCCTCTCCTGAACCTC	—
Bv28_SSR	Failed in singleplex PCR	TAAT	GAGTTAGAAGTGATGCCAATC	GTGTCTGAATACCAGTTGAC	—
Bv38_SS	Failed in singleplex PCR	GATA	GTTTGGTTACAGCCCTCAC	GGTGGTGAAGCTTTCAAATG	—
Bv41_SSR	Failed in singleplex PCR	GATA	GTACAGTCCTAGCAGACCTAG	CAGACCGCACATTACAGG	—
Bv48_SSR	Failed in singleplex PCR	AAAAT	ACCCTAGACCATGAATGTTC	GAGTGATATGAATTTGCAAGG	—

**TABLE A2:** Final set of 46 EPIC primer pairs, including the mix in which we used them for multiplex PCR (Note), the primer sequences (forward and reverse), and the genes they target (gene). The table also inlcudes the two EPIC primers that failed in the multiplex PCRs, as specified in the 'Note' column.

Primer name	Note	Forward	Reverse	Gene
Bv3_EPIC	Mix 1	GTTATCTGCAGGACACGAT	CAGCAGATATCCATGTGAATC	Bromodomain and WD repeat domain containing 1
Bv7_EPIC	Mix 1	GCAACCAACACATCCAATAC	CTGTCATCTTGGGGTCATTC	Potassium channel modulatory factor 1
Bv8_EPIC	Mix 1	GGACAATCATTAGAGGCTAGT	CTTGTTCTGTAGGTGTGCT	Transcription activation suppressor
Bv9_EPIC	Mix 1	ATGGCTCTTGGAGACTACA	TCCTCGGCTCAACATCTC	Eukaryotic translation initiation factor 4A1
Bv10_EPIC	Mix 1	ACATCTGTAAGGTCTGCGA	GGGTGGCAGATTCATAACC	Zic family member 3
Bv11_EPIC	Mix 1	TACGGACCCATCATTTCAAG	CATTTTGCTGCTGTGTATGT	Nudix hydrolase 21
Bv18_EPIC	Mix 1	GAAAAGGTCAAGGAGGGTAA	GGAAGTCCTTCGGTTTCTT	BCL2 associated transcription factor 1
Bv19_EPIC	Mix 1	CTGTTTGCTCAGTTCAAGTC	GTAGAATCTTCAGACCAAGC	SIN3 transcription regulator family member B
Bv23_EPIC	Mix 1	CCACAGCGTCATTAGGTC	CGTCTTCTCCAGCTGTTTC	GATA zinc finger domain containing 2B
Bv27_EPIC	Mix 1	CGTGTGCTTTATGAAGAACG	GCATTTTCTCCAGCTCCTC	Cullin 4B
Bv44_EPIC	Mix 1	TCGGAGGTCTGGGAAAAG	CTGGGTCATTGATGCCATAG	RNA binding motif protein 22
Bv45_EPIC	Mix 1	GAGGTGTCGTAATATCAGTGG	CAAACTCAGTAGGGGTGTAC	DEAF1 transcription factor
Bv2_EPIC	Mix 2	GAAGGCCGTCTTTGATAACC	CTCCCGGACGACATGTAC	Nuclear receptor binding protein 1
Bv5_EPIC	Mix 2	CCACAGCCAGACCATAAC	CTCTCAAAGTTTCGCCTTTC	Pleckstrin homology & coiled‐coil domain containing D1
Bv15_EPIC	Mix 2	GGCCACTTCCTTCTTACTAT	CCAATAGTGAGTGGGATCTC	von Willebrand factor C and EGF domains
Bv24_EPIC	Mix 2	CGAGCAGATGCAGAACTAC	GGATCTTGTCTACTCTTGCG	DENN domain containing 5A
Bv29_EPIC	Mix 2	GATGCGATCACTGCAATG	CTATCAGTGTCTCTTTCCCG	CXXC finger protein 1
Bv31_EPIC	Mix 2	CAGCTTGCAACTCGACTC	CATTAAGGAAGCGGAACTTG	Gamma adaptin ear containing, ARF binding protein 1
Bv32_EPIC	Mix 2	GGAACACAATCCAACCTTCC	GTTTGAACTGCTCCCTTTTC	Pre‐mRNA processing factor 3
Bv39_EPIC	Mix 2	CTCCTGTCCCACTGATCTAC	CCACGTATCCGAAAGACAG	DnaJ heat shock protein family
Bv43_EPIC	Mix 2	GTCTACCATTCTGAGGCTG	CCAGCAACAGGATCAAAAAC	Grainyhead like transcription factor 1
Bv46_EPIC	Mix 2	GCAGAACCTGAAGGAAAAC	GCACTGGTCCTTGAAAATTG	Anti‐Mullerian hormone
Bv47_EPIC	Mix 2	CCTTGGCACACCTGTAATC	GCAGTTTGGGCTACATATTC	von Willebrand factor C and EGF domains
Bv4_EPIC	Mix 3	CAGTCGGCTTTGTTAAATACC	GCAGAATGTTGTCACTCG	Centrosomal protein 85
Bv6_EPIC	Mix 3	CGAGCATCCAAAGTTATCAAG	GGAATCGGGGTAATCCAAG	Syntaxin binding protein 1
Bv12_EPIC	Mix 3	ATATTTACGGGCTCTGGATG	CCCAACAGAGAATATAGGGT	TATA‐box binding protein associated factor 2
Bv13_EPIC	Mix 3	GACGAGTTCGACAAGATGTC	TCCAGCATGATAAACAGGAG	Minichromosome maintenance complex component 3
Bv14_EPIC	Mix 3	GCTTTCTCGACCAGCAAA	CAGTCGATGCCTCAGAATC	Mediator complex subunit 14
Bv25_EPIC	Mix 3	CTAACCGTGGTTCAGCATG	GTCCCTGAGCTGTGACTC	HMG‐box transcription factor 1
Bv28_EPIC	Mix 3	CAGGAAATCGACATCACCAG	GCATAGGCACATCAATTGG	Interferon regulatory factor 1
Bv35_EPIC	Mix 3	CCAGAAGAAAGTCCCTATTCC	CTATCTCTGGACGGGATGAC	Adaptor related protein complex 3 subunit delta 1
Bv36_EPIC	Mix 3	CAAGCAAGACATAAAGGTTGC	GGAGGCAAAAGGTTCTCTG	SOS Ras/Rac guanine nucleotide exchange factor 1
Bv37_EPIC	Mix 3	GACAACTTCAGCAAGATGG	CCACACCAAAACTTTCCAG	Stathmin 1
Bv40_EPIC	Mix 3	GCAACAGAGAGAAATGCTTC	CATTAGCCATGGCATGAATC	Radial spoke head 3
Bv41_EPIC	Mix 3	GAAAGGAGCTGCAGAAGG	CACACAAGTCCACAGGAG	Basic leucine zipper and W2 domains 2
Bv42_EPIC	Mix 3	CAGATGACCTGTCCTCAAC	CTTGGCCCCAAAGATCAG	Nitric oxide synthase 3
Bv1_EPIC	Mix 4	CAAGATGAGGGAGATCGTG	GCAGATCCAAGGTGTAGTTC	WASH complex subunit 5
Bv16_EPIC	Mix 4	TTGACGATATGGATGACTTGG	GCTGAAAGTAGGCAATCTGG	E2F transcription factor 7
Bv17_EPIC	Mix 4	AAGATGAGACCCGTTCTGTA	CCTGTGCCAACATCTTTTG	Eukaryotic translation initiation factor 4E
Bv20_EPIC	Mix 4	CTCGAAGAAGCCATATTCCA	CACCTGCTTGTGCTTGAA	AT‐hook transcription factor
Bv21_EPIC	Mix 4	CTACAATTAACCCTGCCTCTG	CCAGGCTTATGATCTCATCG	Transcription factor binding to IGHM enhancer 3
Bv22_EPIC	Mix 4	GGAAGTGACCTCAAAGAGTAC	GTCCATAGAAGAGGAGAACC	Growth factor independent 1B transcriptional repressor
Bv26_EPIC	Mix 4	CTCAGACTCCCAGCTAAGG	GAGCATTCATCCAGAGCTG	Platelet derived growth factor C
Bv33_EPIC	Mix 4	GATGAGCAAAGAACAAGTGG	AACTCCTCCTGCATTTCTTG	Cingulin
Bv38_EPIC	Mix 4	CAAGTATGGCGTGGAAACC	GTGACTGGACTGTGACCC	Aladin WD repeat nucleoporin
Bv48_EPIC	Mix 4	GTGAACCGAGTGTCTTCC	CAGAGCCTTCTTCTGGTTG	GATA zinc finger domain containing 2B
Bv30_EPIC	Failed in multiplex PCR	CCAACGCACAGAGAGAAATC	CCTCGCTTGCTTCTATTTCC	Raf‐1 proto‐oncogene, serine/threonine kinase
Bv34_EPIC	Failed in multiplex PCR	CACCAAGGAGCAGAAGAC	CAGCTCCCCAATGTCATC	Phosphatidylinositol 4‐kinase beta
