# Supplementary figures and images for: Genotyping by Amplicon Sequencing (GBAS) With Newly Developed SSR and EPIC Markers Reveals Structure in Populations of the Green Toad (Bufotes viridis) Across Rural and Urban Environments
Source: Ecol Evol. 2025 Jul 8;15(7):e71652. doi: 10.1002/ece3.71652 (PMC12235976; doi:10.1002/ece3.71652)

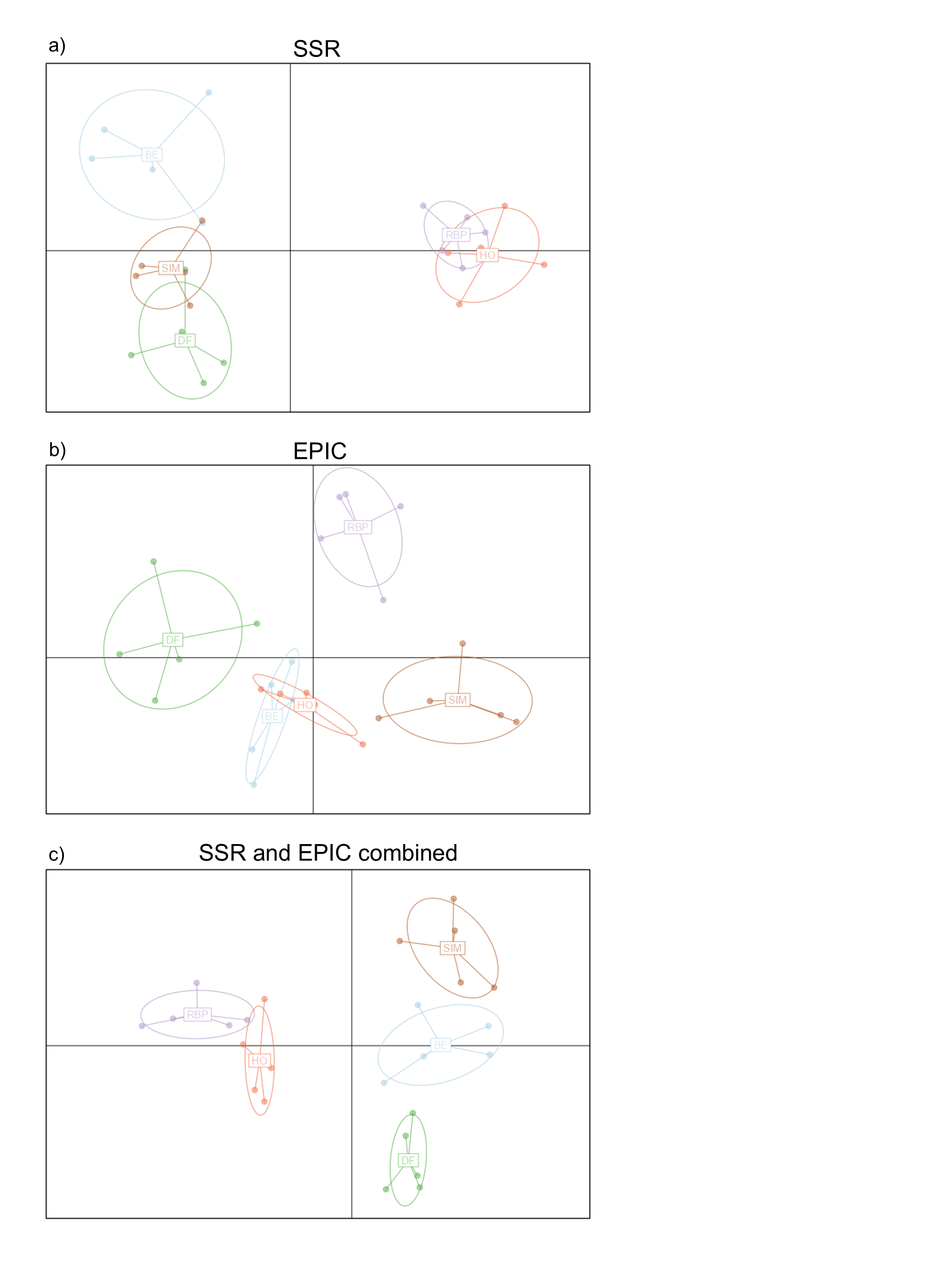

Supplement: Supplementary file 1 — Figure S1. Discriminant analysis of principal components (DAPC) showing all populations downsampled to five individuals. (a) Using only the SSR markers, (b) using only the EPIC markers, (c) using the SSR and EPIC markers combined. BE: Bernhardsthal, DF: Donaufeld, HO: Hohenau, RBP: Rudolf‐Bednar‐Park, SIM: Simmering. [file ECE3-15-e71652-s001.tif]
