# Supplementary material for: Genotyping by Amplicon Sequencing (GBAS) With Newly Developed SSR and EPIC Markers Reveals Structure in Populations of the Green Toad (Bufotes viridis) Across Rural and Urban Environments
Source: Ecol Evol. 2025 Jul 8;15(7):e71652. doi: 10.1002/ece3.71652 (PMC12235976; doi:10.1002/ece3.71652)
Supplement: Supplementary file 2 — Table S1. Test for deviations from Hardy–Weinberg equilibrium (HWE) and values for Wright’s Fixation Index (F IS) per marker, for populations with five or more individuals. [file ECE3-15-e71652-s002.docx]

**Table S1. Test for deviations from Hardy–Weinberg Equilibrium (HWE) and values for Wright’s Fixation Index (F_IS_) per marker, for populations with five or more individuals.** Bv1_SSR to Bv9_SSR are SSR markers. Bv10_EPIC to Bv9_EPIC are EPIC markers. Significant (* p<0.05, ** p<0.01, *** p<0.001) deviations in bold. mono: monomorphic markers, fail: markers that completely failed genotyping.

|  | **Bernhardsthal** | | **Donaufeld** | | **Hohenau** | | **Rudolf-Bednar-Park** | | **Simmering** | |
| --- | --- | --- | --- | --- | --- | --- | --- | --- | --- | --- |
|  | **HWE** | **F_IS_** | **HWE** | **F_IS_** | **HWE** | **F_IS_** | **HWE** | **F_IS_** | **HWE** | **F_IS_** |
| Bv1_SSR | 0.112 | -0.636 | **0.025*** | -0.178 | 0.172 | -0.724 | 0.261 | -0.684 | **0.019*** | -0.665 |
| Bv13_SSR | 0.112 | -0.756 | 0.284 | 0.357 | 0.501 | -0.212 | 0.708 | -0.071 | 0.217 | 0.231 |
| Bv15_SSR | 0.392 | -0.636 | **0.038*** | -0.391 | 0.586 | 0.000 | 0.299 | -0.176 | **0.025*** | -0.028 |
| Bv16_SSR | 0.221 | -0.500 | 0.125 | 0.143 | 0.655 | -0.200 | 0.528 | -0.481 | 0.607 | -0.308 |
| Bv17_SSR | 0.080 | -0.714 | 1.000 | 0.000 | mono | - | 0.804 | -0.111 | 0.473 | 0.179 |
| Bv18_SSR | 0.539 | -0.091 | 0.134 | -0.500 | 0.338 | -0.429 | 0.136 | -0.667 | 0.627 | 0.122 |
| Bv19_SSR | 0.112 | -0.600 | **0.029*** | -0.820 | 0.544 | -0.389 | 0.677 | -0.524 | **0.001***** | -0.641 |
| Bv21_SSR | 0.154 | 0.020 | **0.019*** | 0.100 | 0.338 | -0.429 | 0.338 | -0.429 | **0.000***** | 0.120 |
| Bv22_SSR | 0.459 | -0.200 | 0.062 | -0.266 | 0.994 | -0.333 | 0.577 | 0.000 | **0.014*** | 0.166 |
| Bv24_SSR | mono | - | mono | - | 0.804 | -0.111 | mono | - | 0.908 | -0.151 |
| Bv25_SSR | **0.014*** | 1.000 | **0.003**** | 1.000 | mono | - | mono | - | **0.000***** | 1.000 |
| Bv29_SSR | 0.824 | -0.091 | 0.134 | 0.500 | 0.655 | -0.200 | 0.709 | 0.167 | 0.127 | -0.032 |
| Bv30_SSR | 0.824 | -0.091 | 0.860 | -0.059 | mono | - | mono | - | 0.897 | -0.032 |
| Bv31_SSR | 0.221 | -0.500 | mono | - | 0.576 | -0.250 | 0.804 | -0.111 | 0.248 | 0.289 |
| Bv32_SSR | **0.014*** | -1.000 | 0.249 | -0.385 | **0.025*** | -1.000 | **0.025*** | -1.000 | **0.001**** | -0.362 |
| Bv33_SSR | **0.020*** | 0.444 | **0.027*** | 0.379 | 0.172 | 0.375 | 0.353 | -0.429 | **0.033*** | 0.179 |
| Bv34_SSR | 0.414 | -0.333 | 0.739 | -0.111 | 0.338 | -0.429 | 0.136 | -0.667 | 0.790 | -0.067 |
| Bv35_SSR | 0.414 | -0.333 | 0.391 | -0.286 | 0.804 | -0.111 | 0.241 | 0.524 | 0.356 | -0.231 |
| Bv36_SSR | 0.293 | -0.180 | 0.350 | 0.091 | 0.769 | -0.290 | 0.576 | -0.250 | 1.000 | 0.028 |
| Bv37_SSR | 0.210 | -0.440 | 0.746 | -0.161 | 0.172 | -0.724 | 0.103 | 0.189 | 0.541 | -0.154 |
| Bv39_SSR | 0.371 | -0.241 | **0.019*** | -0.286 | 0.396 | -0.143 | 0.396 | -0.143 | 0.254 | -0.116 |
| Bv4_SSR | **0.007**** | 1.000 | **0.004**** | 0.486 | **0.020*** | 0.444 | 0.247 | -0.220 | **0.000***** | 0.639 |
| Bv43_SSR | 0.824 | -0.091 | 0.249 | -0.385 | 0.709 | 0.167 | 0.338 | -0.429 | 0.270 | 0.203 |
| Bv44_SSR | 0.624 | -0.200 | 0.549 | -0.200 | 0.804 | -0.111 | 0.709 | 0.167 | **0.036*** | -0.524 |
| Bv45_SSR | 0.624 | -0.200 | mono | - | mono | - | mono | - | mono | - |
| Bv46_SSR | 0.644 | -0.364 | **0.019*** | 0.550 | 0.655 | -0.200 | 0.891 | 0.062 | 0.620 | 0.061 |
| Bv47_SSR | 0.624 | -0.200 | 0.391 | -0.286 | 0.709 | 0.167 | 0.241 | 0.524 | 0.611 | 0.127 |
| Bv5_SSR | 0.414 | -0.333 | 0.549 | -0.200 | mono | - | mono | - | 0.897 | -0.032 |
| Bv6_SSR | 0.881 | -0.241 | 0.907 | 0.126 | **0.025*** | -1.000 | 0.576 | -0.250 | 0.810 | -0.008 |
| Bv9_SSR | 0.824 | -0.091 | mono | - | mono | - | mono | - | 0.370 | 0.053 |
| Bv10_EPIC | 0.624 | -0.200 | mono | - | mono | - | mono | - | 0.790 | -0.067 |
| Bv11_EPIC | 0.221 | -0.500 | 0.370 | 0.299 | 0.775 | -0.143 | 1.000 | 0.000 | 0.459 | -0.185 |
| Bv12_EPIC | 0.414 | -0.333 | 0.722 | -0.268 | mono | - | 0.804 | -0.111 | 0.685 | -0.133 |
| Bv13_EPIC | 0.995 | -0.200 | 0.970 | -0.250 | 0.386 | -0.500 | 0.775 | -0.143 | 0.248 | 0.048 |
| Bv15_EPIC | 0.253 | -0.600 | 0.478 | 0.349 | 0.135 | 0.286 | 0.655 | -0.200 | 0.128 | -0.139 |
| Bv16_EPIC | 0.944 | -0.029 | 0.549 | -0.200 | 0.136 | -0.667 | mono | - | **0.012*** | -0.631 |
| Bv17_EPIC | mono | - | 0.860 | -0.059 | mono | - | mono | - | mono | - |
| Bv18_EPIC | mono | - | 0.860 | -0.059 | mono | - | mono | - | mono | - |
| Bv19_EPIC | 0.624 | -0.200 | mono | - | mono | - | mono | - | 0.612 | -0.025 |
| Bv1_EPIC | 0.531 | -0.224 | 0.257 | -0.371 | 0.261 | 0.200 | 0.230 | -0.600 | 0.851 | -0.193 |
| Bv20_EPIC | mono | - | 0.612 | 0.169 | 0.637 | -0.333 | 0.351 | 0.467 | 0.341 | 0.238 |
| Bv21_EPIC | 0.080 | -0.714 | mono | - | 0.576 | -0.250 | 0.576 | -0.250 | 0.743 | -0.082 |
| Bv22_EPIC | 0.291 | -0.021 | 1.000 | 0.000 | **0.019*** | 0.412 | 0.136 | -0.667 | 0.770 | -0.311 |
| Bv23_EPIC | 0.382 | -0.463 | 0.931 | -0.091 | 0.931 | -0.231 | 0.230 | -0.600 | **0.026*** | 0.082 |

**Table S1. (continued).**

|  | **Bernhardsthal** | | **Donaufeld** | | **Hohenau** | | **Rudolf-Bednar-Park** | | **Simmering** | |
| --- | --- | --- | --- | --- | --- | --- | --- | --- | --- | --- |
|  | **HWE** | **F_IS_** | **HWE** | **F_IS_** | **HWE** | **F_IS_** | **HWE** | **F_IS_** | **HWE** | **F_IS_** |
| Bv24_EPIC | 0.682 | -0.371 | 0.860 | -0.059 | 0.223 | 0.130 | 0.769 | -0.290 | 0.568 | 0.003 |
| Bv25_EPIC | 0.221 | -0.500 | 0.134 | 0.500 | 0.709 | 0.167 | mono | - | 0.341 | 0.238 |
| Bv26_EPIC | **0.014*** | -1.000 | **0.016*** | -0.800 | fail | - | **0.046*** | -1.000 | **0.000***** | -1.000 |
| Bv27_EPIC | **0.029*** | 0.333 | 0.125 | 0.310 | 0.423 | 0.077 | **0.019*** | 0.091 | **0.000***** | 0.058 |
| Bv28_EPIC | mono | - | 0.613 | -0.169 | mono | - | mono | - | mono | - |
| Bv29_EPIC | mono | - | mono | - | mono | - | mono | - | 0.790 | -0.067 |
| Bv2_EPIC | 0.174 | 0.556 | 0.549 | -0.200 | 0.223 | 0.130 | 0.338 | -0.429 | 0.536 | 0.264 |
| Bv31_EPIC | 0.971 | -0.143 | 0.865 | -0.220 | 0.789 | -0.034 | **0.025*** | 1.000 | 0.790 | 0.224 |
| Bv32_EPIC | 0.414 | -0.333 | 0.764 | 0.100 | 0.709 | 0.167 | 0.338 | -0.429 | 0.459 | -0.185 |
| Bv33_EPIC | 0.624 | -0.200 | 0.739 | -0.111 | mono | - | mono | - | 0.955 | -0.103 |
| Bv35_EPIC | 0.944 | -0.029 | 1.000 | 0.000 | 0.180 | 0.600 | 0.338 | -0.429 | 0.069 | -0.455 |
| Bv36_EPIC | 0.414 | -0.333 | 1.000 | 0.000 | mono | - | 0.484 | 0.310 | 0.679 | -0.103 |
| Bv37_EPIC | 0.414 | -0.333 | 0.860 | -0.059 | 0.709 | 0.167 | **0.019*** | 0.412 | 0.356 | -0.070 |
| Bv38_EPIC | 0.824 | -0.091 | mono | - | mono | - | mono | - | mono | - |
| Bv39_EPIC | 0.881 | -0.241 | 0.116 | -0.034 | mono | - | 0.821 | -0.304 | 0.991 | -0.164 |
| Bv3_EPIC | 0.221 | -0.500 | 0.887 | 0.065 | 0.386 | -0.500 | **0.025*** | 1.000 | 0.053 | 0.354 |
| Bv40_EPIC | 0.095 | -0.469 | **0.006**** | -0.286 | fail | - | 0.217 | 0.579 | **0.000***** | 0.388 |
| Bv41_EPIC | 0.073 | 0.122 | 0.692 | -0.059 | 0.505 | -0.333 | 0.506 | -0.263 | 0.686 | 0.167 |
| Bv42_EPIC | 0.054 | 0.027 | 0.860 | -0.059 | 0.349 | 0.474 | 0.338 | -0.429 | 0.883 | -0.210 |
| Bv43_EPIC | mono | - | 0.612 | 0.169 | mono | - | mono | - | 0.897 | -0.032 |
| Bv44_EPIC | 0.881 | -0.116 | 0.860 | -0.059 | mono | - | 0.804 | -0.111 | 0.177 | -0.170 |
| Bv45_EPIC | 0.624 | -0.200 | 0.722 | -0.268 | 0.692 | 0.143 | 0.338 | -0.429 | 0.277 | 0.295 |
| Bv47_EPIC | mono | - | mono | - | mono | - | mono | - | mono | - |
| Bv4_EPIC | 0.414 | -0.333 | 0.708 | -0.125 | 0.989 | -0.250 | **0.025*** | 1.000 | **0.003**** | 0.733 |
| Bv5_EPIC | 0.414 | -0.333 | 0.302 | -0.518 | 0.576 | -0.250 | mono | - | 0.790 | -0.067 |
| Bv6_EPIC | mono | - | **0.035*** | 0.213 | fail | - | 0.351 | 0.467 | 0.114 | 0.228 |
| Bv7_EPIC | 0.824 | -0.091 | 0.708 | -0.125 | 0.804 | -0.111 | 0.804 | -0.111 | 0.563 | -0.042 |
| Bv8_EPIC | mono | - | 0.708 | -0.125 | mono | - | mono | - | 0.790 | -0.067 |
| Bv9_EPIC | 0.414 | -0.333 | 0.284 | 0.357 | 0.637 | -0.333 | 0.351 | 0.467 | 0.083 | 0.102 |
